# Machine learning to predict post-operative acute kidney injury stage 3 after heart transplantation

**DOI:** 10.1186/s12872-022-02721-7

**Published:** 2022-06-25

**Authors:** Tingyu Li, Yuelong Yang, Jinsong Huang, Rui Chen, Yijin Wu, Zhuo Li, Guisen Lin, Hui Liu, Min Wu

**Affiliations:** 1 Guangdong Cardiovascular Institute, Guangdong Provincial Key Laboratory of South China Structural Heart Disease, Guangdong Provincial People’s Hospital, Guangdong Academy of Medical Sciences, 106 Zhongshan 2nd Road, Guangzhou, 510080 Guangdong China; 2 The Second School of Clinical Medicine, Southern Medical University, Guangzhou, Guangdong China; 3 Department of Radiology, Guangdong Provincial People’s Hospital, Guangdong Academy of Medical Sciences, Guangzhou, Guangdong China; 4 Department of Nephrology, Guangdong Provincial People’s Hospital, Guangdong Academy of Medical Sciences, Guangzhou, Guangdong China

**Keywords:** Acute kidney injury, Heart transplantation, Predictive model, Machine learning

## Abstract

**Background:**

Acute kidney injury (AKI) stage 3, one of the most severe complications in patients with heart transplantation (HT), is associated with substantial morbidity and mortality. We aimed to develop a machine learning (ML) model to predict post-transplant AKI stage 3 based on preoperative and perioperative features.

**Methods:**

Data from 107 consecutive HT recipients in the provincial center between 2018 and 2020 were included for analysis. Logistic regression with L2 regularization was used for the ML model building. The predictive performance of the ML model was assessed using the area under the curve (AUC) in tenfold stratified cross-validation and was compared with that of the Cleveland-clinical model.

**Results:**

Post-transplant AKI occurred in 76 (71.0%) patients including 15 (14.0%) stage 1, 18 (16.8%) stage 2, and 43 (40.2%) stage 3 cases. The top six features selected for the ML model to predicate AKI stage 3 were serum cystatin C, estimated glomerular filtration rate (eGFR), right atrial long-axis dimension, left atrial anteroposterior dimension, serum creatinine (SCr) and FVII. The predictive performance of the ML model (AUC: 0.821; 95% confidence interval [CI]: 0.740–0.901) was significantly higher compared with that of the Cleveland-clinical model (AUC: 0.654; 95% [CI]: 0.545–0.763, *p* < 0.05).

**Conclusions:**

The ML model, which achieved an effective predictive performance for post-transplant AKI stage 3, may be helpful for timely intervention to improve the patient’s prognosis.

**Supplementary Information:**

The online version contains supplementary material available at 10.1186/s12872-022-02721-7.

## Background

Heart transplantation (HT) remains as a life-sustaining treatment choice for numerous end-stage heart disease patients [1]. Despite the advancement of various immunosuppressive therapies and treatment programs, the incidence rates of acute kidney injury (AKI) as well as severe AKI requiring renal replacement therapy (RRT) in patients with HT remain high in recent years [2]. AKI most commonly occurs in the first week after HT, with the incidence of 22–76%, and is associated with high rates of morbidity and mortality [3–6].

Early AKI detection after HT is meaningful for interventions that prevent future kidney damage and preserve the kidney function, because AKI is associated with more than 60% mortality rate among hospitalized postsurgical patients who received intensive care [7]. Moreover, AKI, especially stage 3, is correlated with subsequent progressive chronic kidney disease (CKD) along with decreased survival rates of HT recipients. Various features associated with AKI stage 3, such as drugs, immunosuppression therapies, hemodynamics, and some anesthesia- and surgery-related factors have been identified by traditional models in previous studies [6, 8]. However, their predictive performance is relatively limited due to the limited amount of patient’s information extracted and some unsatisfying features with conflicting effects. For these reasons, it is indispensable to develop a novel and efficient model to predict AKI stage 3.

As a powerful tool for intelligent data analysis, machine learning (ML) can be utilized to model medical data. Computational algorithms are constructed to develop a model to correlate a spectrum of features of the given datasets with the outcome. ML has been commonly used in medical data analysis for diagnosis and prognosis of a variety of tumors, such as breast [9] and prostate cancers [10]. Furthermore, there is clear evidence that ML can be used for analysis in other medical fields as well. For instance, a recent study on predicting 5-year all-cause mortality in patients with suspected coronary artery disease showed that ML had superior predictive performance compared with traditional clinical or coronary computed tomography angiography metrics alone [11]. We hypothesize that ML adds incremental value to the prediction of adverse events. Therefore, the objective of this study was to evaluate the feasibility and accuracy of ML to predict AKI stage 3 in HT patients and then to compare the performance to that of existing clinical metrics.

## Methods

### Data collection

**Fig. 1:**
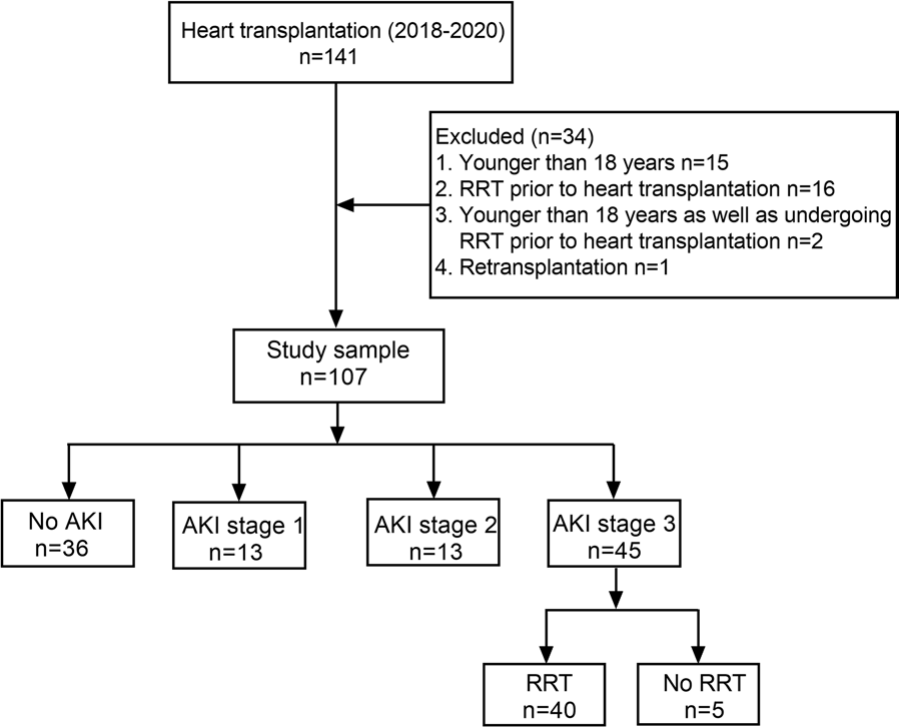
Diagram of study population based on AKI severity postoperatively. AKI, acute kidney injury; RRT, renal replacement therapy

Data of all HT patients in Guangdong Provincial People's Hospital were collected and analyzed from January 2018 through September 2020. All patients had undergone primary orthotopic deceased-donor HT due to various causes. Exclusion criteria were recipient age < 18 years at the time of operation, retransplantation, or RRT prior to HT (Fig. 1). We obtained patient data from the hospital database or electronic records. This retrospective study was approved by the Institutional Review Board of the Guangdong Provincial People's Hospital and was conducted in accordance with the Declaration of Helsinki. The need for informed consent was waived given the retrospective nature of the study.

### Study features

We reviewed the patients’s medical records retrospectively and collected clinical data including: demographic features, pretransplant renal function features, liver function features, the use of invasive hemodynamic support therapies, echocardiography features, donor characteristics, aortic clamp time, cardiopulmonary bypass time, blood transfusion. All features were divided into three subsets: preoperative, perioperative, and donor characteristics.

### Study outcomes

The study outcome was post-transplant AKI defined based on the Kidney Disease: Improving Global Outcomes (KDIGO) criteria [12]: an increase in serum creatinine (SCr) by ≥ 0.3 mg/dl (≥ 26.5 umol/L) within 48 h or to > 1.5 times baseline within the first 7 postoperative days. AKI was classified into 3 stages depending on the level of SCr: stage 1, SCr increase by ≥ 0.3 mg/dl (≥ 26.5 umol/L) within 48 h or 1.5–1.9-fold increase from the baseline; stage 2, 2–2.9-fold increase from the baseline; stage 3, ≥ threefold increase from the baseline, increase in SCr by ≥ 4.0 mg/dl (≥ 354 umol/L) or the start of RRT. The baseline SCr was referred to the last SCr value before HT. Next, we calculated the estimated glomerular filtration rate (eGFR) by using the Chronic Kidney Disease-Epidemiology Collaboration Group equation [13].

### Machine learning

**Fig. 2:**
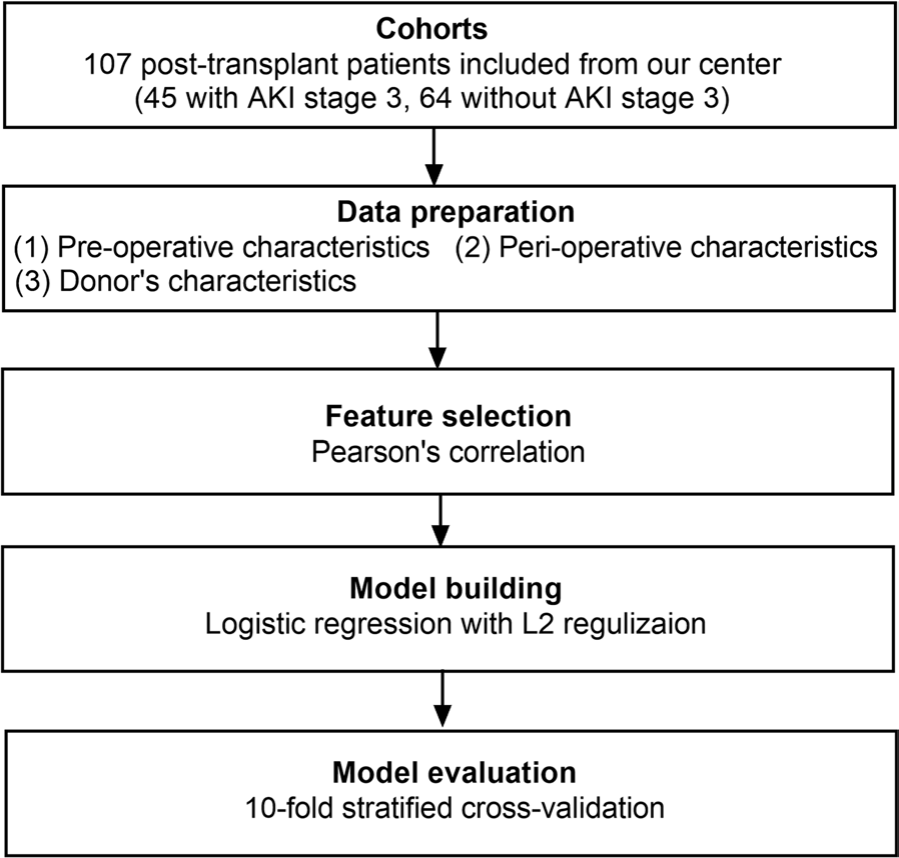
Workflow for the classification of patients undergoing HT with and without post-transplant AKI stage 3 using machine learning. AKI, acute kidney injury

Feature selection, model building, and model evaluation were all part of the ML system (Fig. 2). It was mostly implemented in WEKA 3.8.

**Fig. 3:**
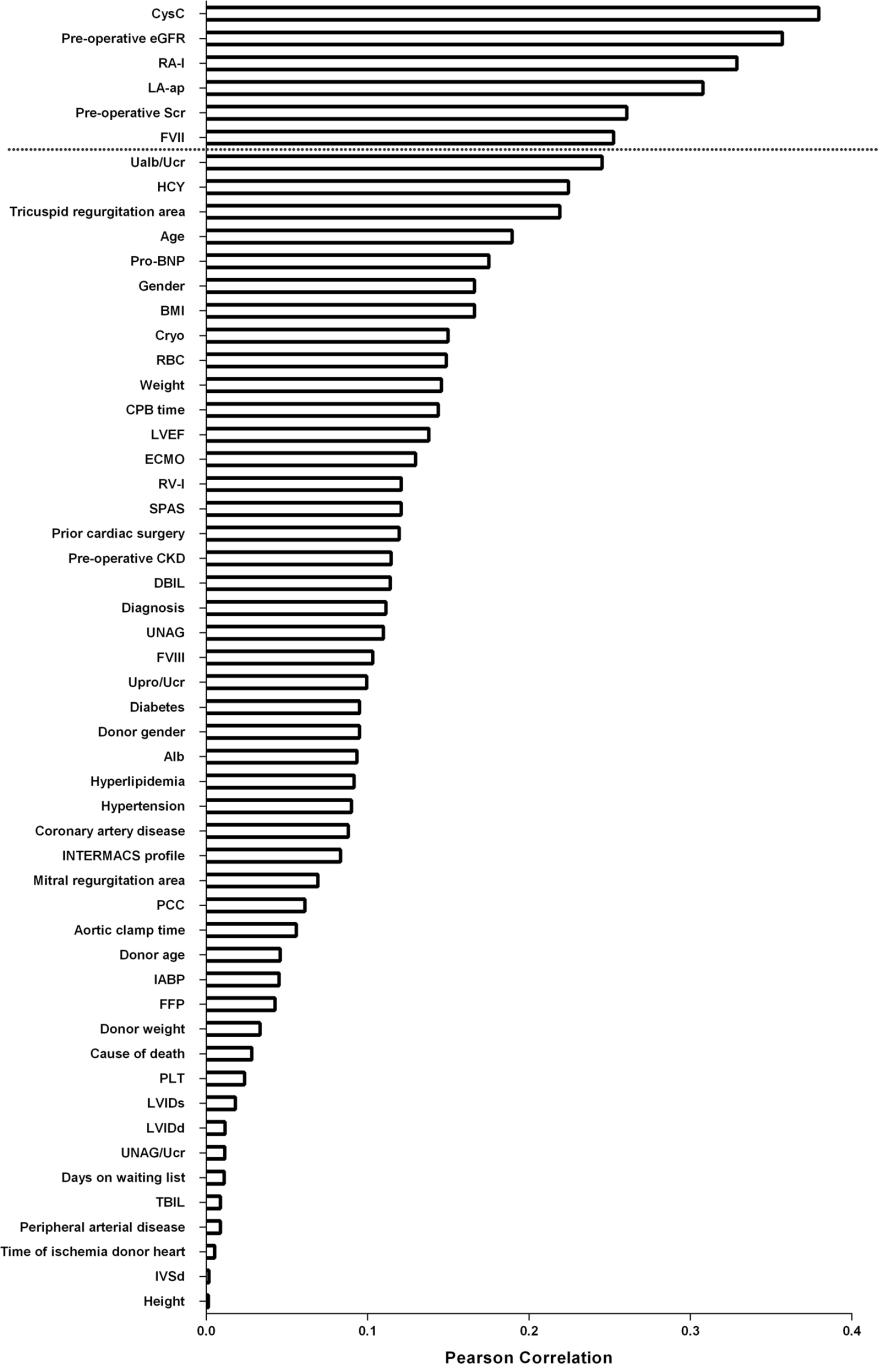
Feature selection. Pearson's correlation was used to evaluate the worth of a feature. The features were ranked in descending order by Pearson's correlation and the top six features were used to build the ML model. Abbreviation as in Table 1

To evaluate the worth of a feature, Pearson's correlation between the feature and the class was calculated. The correlations between the features included were ranked descendingly (Fig. 3), and we selected those most closely related to postoperative AKI stage 3. The details of feature selection are shown in the additional file: (see Additional file 1: Table S1).

We selected different classifiers (e.g., logistic regression with L2 regularization, logistic regression, random forest, naïve Bayes, and support vector machine) to build classifiers based on the features highly correlated with AKI stage 3. Detailed information about the model selection is available in the additional file: (see Additional file 2: Table S1). After the model selection procedure, the logistic regression with L2 regularization was the selected model. L2 regularization is a regularized method that shrinks the regression coefficients towards 0 by placing a penalty on the summation of the estimated coefficients. Although the regularization method may lead to biased regression estimates, it results in a more stable model that produces excellent predictive performance in particular when applied to external datasets (further details about the algorithm are available in the Additional file 3) [14].

A tenfold cross-validation was used to assess the performance of the ML model. The dataset was randomly divided into ten folds with approximately the same number of patients in each fold. Nine folds served as the training set, while the remaining fold served as the validation set. In all, each fold was used nine times as a training set and once as a validation set. Thus, the outcome of each patient was predicted once.

### Statistical analysis

Continuous features with normal distribution based upon the Durbin-Watson test were presented as mean ± standard deviation; data with skewed distributions were presented as median and interquartile range (IQR); and categorical features were presented as frequency (percentage). The receiver operator characteristic curves were used to evaluate the performance of the ML model and of the reported Cleveland-clinical model to predict post-transplant AKI stage 3. In Cleveland-clinical model, preoperative serum creatinine level, serum albumin level, insulin-requiring diabetes, and cardiopulmonary bypass time have been reported based on large samples study as independent predictors of postoperative AKI [15]. And the differences between areas under the curves (AUCs) were compared based on Delong et al. [16]. The accuracy, sensitivity, and specificity of the model based on the optimum cutoffs were computed. All statistical analyses were performed with SPSS version 22.0 software (SPSS, Chicago, Illinois, USA) and R statistical software (R Foundation, Vienna, Austria) by using RStudio Server version 1.3. The presented statistical significance levels were all two-sided and *p* < 0.05 was considered significant.

## Results

### Study population

From 141 patients with HT from January 2018 through September 2020, 34 were excluded for the following reasons: younger than 18 years old (n = 15); had preoperative RRT (n = 16); younger than 18 years old and had preoperative RRT (n = 2); and had retransplantation (n = 1). Finally, a sample of 107 patients was analyzed with 76 (71.0%) patients suffering from AKI. Furthermore, the incidences of AKI stages 1, 2, and 3 were 15 (14.0%), 18 (16.8%), and 43 (40.21%), respectively. Of those who met the criteria for AKI stage 3, 40 (93.0%) received RRT, which lasted for a median of 159 (76–373) hours, and 18 (41.8%) deaths were observed (Table 1). Donor, recipient, and surgery-related characteristics are listed in Table 1.

**Table 1:** Donor, recipient and surgical characteristics in the cohorts

Features	No AKI 31 (28.9)	AKI stage1 15 (14.0)	AKI stage2 18 (16.8)	AKI stage3 43 (40.2)
**Preoperative characteristics**				
Age, years	49 (37–60)	48 (29–57)	47 (32–55)	52 (46–59)
Male, sex	26 (83.9)	12 (80)	14 (77.8)	40 (93.0)
Height, cm	168 (162–172)	168 (165–170)	169 (163–175)	168 (163–172)
Weight, kg	58 (53–70)	60 (54–69)	65 (52–70)	62 (55–77)
BMI, kg/m^2^	21 (19–24)	20 (19–24)	22 (20–24)	23 (20–27)
Serum albumin	41.2 (37.5–43.7)	40.9 (38.5–42.6)	42.4 (38.8–44.3)	39.2 (36.8–42.5)
**Primary cardiac disease (yes)**				
Dilated cardiomyopathy	21 (67.7)	11 (73.3)	7 (38.8)	22 (51.2)
Valvular disease	3 (9.7)	0 (0)	1 (5.6)	8 (18.6
Ischemic cardiac disease	4 (3.2)	2 (13.3)	6 (33.3)	11 (25.6)
Restrictive cardiomyopathy	1 (3.2)	1 (6.7)	0 (0.0)	11 (25.6)
Hypertrophic cardiomyopathy	1 (3.2)	0 (0.0)	1 (5.6)	1 (2.3)
Other cardiac disease	1 (3.2)	1 (6.7)	2 (11.1)	5 (11.6)
**Medical history (yes)**				
Prior cardiac surgery	7 (22.6)	8 (53.3)	10 (55.6)	
Insulin-requiring diabetes	1 (3.2)	1 (6.6)	2 (11.1)	8 (18.6)
Hypertension	3 (9.7)	2 (13.3)	7 (6.5)	9 (20.9)
Hyperlipidemia	0 (0.0)	0 (0.0)	1 (5.6)	2 (4.7)
Peripheral vascular disease	4 (12.9)	0 (0.0)	6 (33.3)	7 (16.3)
Coronary arterial disease	4 (12.9)	2 (13.3)	7 (38.9)	12
**Heart function**				
NT-proBNP, pg/mL	1982 (1083–3848)	4076 (1350–6001)	2498 (1031–4513)	4328 (1223–10,506)
HCY, μmol/L	10 (7–40)	6 (4–6)	12 (8–12)	257 (10–897)
**INTEMACAS**				
1	2 (6.5)	0 (0.0)	0 (0.0)	1 (2.3)
2	1 (3.2)	2 (13.3)	0 (0.0)	1 (2.3)
3	1 (3.2)	1 (6.7)	0 (0.0)	4 (9.3)
4	3 (9.7)	3 (20.0)	5 (27.8)	5 (11.6)
5	1 (3.2)	0 (0.0)	0 (0.0)	6 (14.0)
6	6 (19.4)	3 (20.0)	2 (11.1)	7 (16.3)
7	17 (54.8)	6 (40)	11 (61.1)	19 (44.2)
**Renal function**				
Baseline SCr, mmol/L	98 (78–118)	77 (71–87)	85 (71–85)	109 (91–150)
eGFR, ml/min/1.73m^2^	78 (63–93)	102 (77–112)	89 (76–113)	64 (41–83)
CKD (eGFR < 60 mL/min per 1.73 m^2^) (yes)	6 (19.3)	0 (0.0)	0 (0.0)	16 (37.2)
UNAG, U/L	17 (10–38)	17 (7–40)	14 (12–25)	25 (8–43)
UNAG/Ucr, U/mmol	5 (2–21)	4 (1–13)	3 (2–17)	10 (3–26)
CysC, mg/L	1.4 (0.9–1.7)	1.0 (0.8–1.5)	1.1 (0.9–1.4)	1.6 (1.4–2.5)
Ualb/Ucr, mg/g	122 (87–182)	129 (35–275))	93 (9–169)	160 (28–1139)
Upro/Ucr, mg/g	156 (38–295)	106 (14–11,620)	61 (53–61)	148 (19–312)
**Liver function**				
TBIL, umol/L	20 (15–34)	21 (19–25)	19 (15–23)	19 (14–29)
DBIL, umol/L	5 (3–10)	5 (4–8)	4 (3–5)	5 (3–10)
**Preoccupative support (yes)**				
IABP	5 (16.1)	2 (13.3)	0 (0.0)	6 (14.9)
ECMO	2 (6.5)	3 (20.0)	2 (11.1))	4 (9.3)
**Echocardiography**				
LA-ap, mm	46 (40–53)	52 (45–55)		53 (45–60)
LVIDd, mm	71 (60–78)	73 (65–89)	66 (59–72)	67 (62–77)
LVIDs, mm	63 (50–69)	66 (53–78)	58 (50–67)	59 (51–70)
LVEF, %	26 (23–31)	23 (18–33)	27 (23–34)	24 (19–32)
RV-l, mm	61 (52–57)	60 (47–68)	61 (57–64)	63 (58–71)
RA-l, mm	51 (46–58)	60 (47–68))	50 (46–58)	63 (54–59)
IVSd, mm	9 (7–10)	8 (7–9)	9 (8–9)	9 (8–10)
Mitral regurgitation area, cm^2^	7 (3–12)	8 (4–14)	4 (2–9)	8 (3–11)
Tricuspid regurgitation area, cm^2^	3 (1–6)	6 (1–8)	3 (1–4)	6 (3–9)
SPAP, mmHg	34 (27–58)	38 (31–59)	45 (31–55)	46 (38–60)
Days on waiting list	21 (11–30)	21 (18–30)	24 (10–56)	21 (7–33)
**Donor characteristics**				
Age, years	37 (31–43)	33 (22–44)	47 (32–54)	33 (23–45)
Male, sex	29 (93.5)	14 (93.3)	17 (94.4)	38 (88.4)
Weight, kg	65 (60–68)	60 (57–70)	65 (52–70)	60 (65–70)
**Cause of death (yes)**				
Trauma	21 (67.7)	10 (66.7)	10 (55.6)	28 (65.1)
CVA	9 (29.0)	4 (26.7)	6 (33.3)	11 (25.6)
Others	4 (3.2)	5 (6.7)	2 (11.1)	4 (9.3)
Time of ischemia donor heart, min	202 (183–234)		219 (171–283)	207 (180–256)
**Perioperative characteristics**				
Aortic clamp time, min	131 (110–139)	123 (118–134)	133 (113–162)	122 (113–144)
CPB time, min	250 (219-312C)	250 (219–312)	261 (210–298)	245 (225–293)
**Intra-op transfusions—units**				
FVII	400 (400–400)	200 (200–200)	/	200 (200–200)
FVIII	800 (800–800)	800 (800–800)	800 (800–800)	800 (800–800))
PCC	800 (800–800)	800 (800–800)	800 (800–800)	800 (800–800)
Cryo	10 (10–10)	10 (10–11)	10 (10–10)	10 (10–10)
RBC	6 (2–6)	3 (2–3)	3.5 (3.5–3.5)	6 (4–8)
PLT	1 (1–2)	1 (1–2)	1 (1–2)	1 (1–1)
FFP	400 (0–400)	400 (400–400)	400 (400–400)	400 (400–600)
**Postoperative characteristics**				
Post-operative SCr, μmol/L	128 (95–147)	135 (119–150)	206 (153–255)	347 (225–467)
RRT (yes)	0 (0.0)	0 (0.0)	0 (0.0)	40 (93.0)
RRT time, h	0 (0–0)	0 (0–0)	0 (0–0)	159 (76–373)
Days in ICU	6 (5–8)	6 (5–9)	7 (6–12)	10 (8–16)
Re-admission to hospital (yes)	3 (9.7)	2 (13.3)	2 (11.1)	11 (25.6)
Death (yes)	6 (19.4)	3 (20.0)	0 (0.0)	18 (41.8)

Data displayed as median and interquartile range or n (%)

BMI, body mass index; CKD, chronic kidney disease; CPB, cardiopulmonary bypass; Cryo, cryoprecipitation; CVA, cerebrovascular accident; CysC, cystatin C; DBIL, direct bilirubin; ECMO, extracorporeal membrane oxygenator; eGFR, estimated glomerular filtration rate; FFP, fresh frozen plasma; FVII, factor VII; FVIII, factor VIII; HCY, homocysteine; IABP, intra-aortic balloon pump; ICU, intensive care unit; IVSd, interventricular septal end-diastolic thickness; LA-ap, left atrial anteroposterior dimension; LVEF, left ventricular ejection fraction; LVIDd, left ventricular internal diameter in diastole; LVIDs, left ventricular internal diameter in systole; NT-proBNP, N-terminal pro brain-type natriuretic peptide; PCC, prothrombin complex concentrate; RA-l, right atrial long-axis dimension; RBC, red blood cell; RRT, renal replacement therapy; RV-l, right ventricular long-axis dimension; SCr, serum creatinine; SPAP, systolic pulmonary artery pressure; TBIL, total bilirubin; UAlb, urine albumin; Ucr, urine creatinine; PLT, blood platelet; UNAG, urine N-acetyl-κ-d-glucosaminidas; Upro, urine protein

### Feature selection

The features were ranked by Pearson correlation in descending order (Fig. 3). The top six features were identified as significant and used to train the ML model. Those features were as follows: preoperative serum CysC (r = 0.379), eGFR (r = 0.357), right atrial long-axis dimension (RA-l; r = 0.328), left atrial anteroposterior dimension (LA-ap, r = 0.307), SCr (r = 0.260), and FVII (r = 0.252).

### Prediction of AKI stage 3

**Fig. 4:**
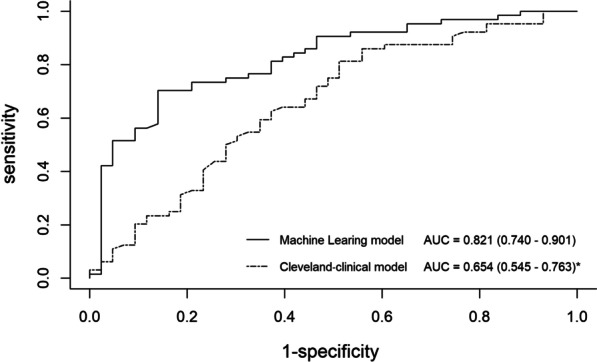
Receiver operating characteristic curves for prediction of post-transplant AKI stage 3. For AKI stage 3 prediction, machine learning using the logistic regression with L2 regularization in tenfold cross-validation showed a significantly higher area under the curve than all other clinical metrics using DeLong's test (*p < 0.05, **p < 0.001). AUC, area under the curve; ML, machine learning;

The ML model exhibited a significantly higher AUC (0.821; 95% [CI]: 0.740–0.901) compared to the existing clinical model for prediction of AKI stage 3 (AUC: 0.654, 95% [CI]: 0.545–0.763, *p* < 0.05) (Fig. 4). The accuracy, sensitivity, and specificity for the prediction of AKI stage 3 were 80.4%, 86.0%, and 70.3% for the ML model; and 69.2%, 44.2%, and 41% for Cleveland clinical model, respectively.

## Discussion

In the present study, the results suggested that the ML model could be an effective tool for risk stratification and prediction of post-transplant AKI stage 3 for individual patients. The performance of the ML model was superior to that of the reported clinical model confirmed by large samples at the Cleveland Clinic Foundation. As far as we know, this study is the first to evaluate the predictive capability of ML methods for the assessment of severe postoperative AKI in patients undergoing HT.

Early identification and prevention of AKI in patients undergoing HT may play an important role in selecting treatment regimens and thus improving prognosis, given the high short- and long-term mortality risks associated with AKI after HT. If acute renal failure happens, the short-term mortality increases 3.5-fold and 1-year mortality 2.3-fold [1]. However, the ability to accurately identify high-risk patients who may develop AKI is a major challenge in clinical practice. Although traditional risk factors for the prediction of post-transplant AKI have been identified, they are population-based tools [7, 17], which are less effective for individual risk evaluations. Furthermore, the traditional features to predict post-transplant AKI from existing models have relatively limited predictive performance [18], highlighting the need for a more precise model for personalized treatment decisions.

Analyzing and integrating numerous risk features in each individual patient can be a challenging task for the clinician. The increasing number of clinical features affecting risk stratification from various medical checks amplifies the intricacy of assessment and makes it more difficult for clinicians to make a correct decision involving risk stratification in each patient. Moreover, the unanticipated aspects of possible interactions between a few weaker risk features in an individual patient are frequently underestimated [11]. Machine learning, both supervised and unsupervised, can overcome these challenges by deep integration of the experimental and clinical datasets to build powerful risk models and reclassify patient groups [19].

Our results demonstrated that by the integration of clinical information, experimental datasets, and ultrasonography-derived metrics, the ML model (AUC: 0.821) showed superior risk prediction for AKI stage 3 compared with Cleveland-clinical model (AUC: 0.654). The features had been identified as predictors of AKI by logistic regression analysis in previous studies [15]. In our study, the ML model provided an excellent value in prognostic performance while considering 53 features and potential feature–feature interactions in patients. This characteristic permits a deep exploration of all available data for non-linear patterns that could predict the risk stratification of a particular individual [14].

As reported in previous studies, the occurrence of AKI is the consequence of multifactorial interactions that cannot be interpreted with a single etiologic factor [18, 20, 21]. In the light of our findings, CysC, eGFR, RA-l, LA-ap, SCr and FVII were all predictive factors included in the ML model for predicting the development of AKI stage 3. In particular, CysC, a biomarker for the quantification of kidney function loss, was the most related predictive factor in patients with AKI stage 3, and it may have the ability to detect AKI one to two days before the rise of SCr with higher accuracy and precision [22]. Furthermore, except for acute renal failure, no other factors were found to alter CysC levels, enhancing its effectiveness as an endogenous marker for predicting AKI. Our findings confirm the predictive value of eGFR ranked lower than CysC. One explanation for this may be that CysC reflects GFR changes more sensitively compared to SCr, and eGFR, which used widely in clinical practice instead of GFR, is calculated with SCr in this study[22].

Cardiac features can reflect the confluence of heart–kidney interactions through hemodynamic dimensions. The difference between arterial perfusion pressure and venous outflow pressures must be adequately large to keep sufficient renal blood flow and glomerular filtration. In the setting of this concept, the inability of impaired left ventricular function makes low forward flow with reduced left ventricular ejection fraction (LVEF), and consequently leading to prerenal hypoperfusion. Interestingly, we found that LVEF had no significant effect on the development of AKI stage 3. This is supported by previous studies, such as Jin et al. [23] demonstrated that LVEF was not independently or significantly associated with the development of AKI after cardiac operations. This was illustrated by a relative preservation of eGFR derived from efferent arteriolar constriction following on from the renin-angiotensin system to accommodate the decreased LVEF. In patients with markedly reduced renal blood flow exceeding renal autoregulatory capacity, the compensatory increase in eGFR was lost and could evolve into AKI. Alternatively, the elevated central venous pressures, as a result of changes in right heart structure such as an augmented diameter of RA-l, can bring about an increased renal resistance; the kidneys may subsequently become more susceptible to the occurrence of AKI. This mechanism has been presented in clinical researches in patients with cardiac dysfunction using invasive hemodynamic measurements [24, 25].

The relationship between coagulation factors and the incidence of AKI should be further verified by large samples. FVII was turned out to be a predictor of post-transplant AKI in this study, although consistent with other prior work that higher numbers of transfusions, particularly higher blood and cryoprecipitate transfusion, were associated with the incidence of AKI [26]. However, In Jocher et al., there were no differences in the intra-op pRBC, FFP, platelets, or coagulation factors between the No-AKI and AKI groups, suggesting that transfusion was not a risk factor of AKI [27]. The decision to transfuse is influenced by unmeasured factors, such as severity of intraoperative bleeding and pre-existing comorbidities.

There was a high incidence of AKI (71%) in this study, which met the upper end of the incidence range of 22–76% reported in prior studies [3–6]. We speculated that there may be the following reasons. Our cohort had a long CPB duration that was associated with a higher incidence of post-operative AKI [26–28]. Several mechanisms may play crucial roles, including renal hemodynamic changes (hemodilution, hypothermia, and non-pulsatile flow), hemolysis caused by turbulent flow and occlusive roller pumps leading to generation of reactive oxygen species [21]. In addition, most of our patients were admitted to hospital for acute heart failure, especially the incidence of right heart failure was relatively high. And RV function is a central determinant of Cardiorenal Syndrome hemodynamics [20]. Patients after HT underwent intrinsic oxidative stress as well as systematic and intrarenal inflammation, which is related to AKI [29, 30]. This could be explained by renal tubular epithelial cells are extremely susceptible to oxidative stress, particularly during ischemia–reperfusion phase.

### Study limitations

This study has several limitations. First, our research was a single center study with a relatively limited sample size. Although the ridge logistic regression with the L2 regularization could cope with over-fitting problems that may occur owing to small sample size, a multicenter study will be better to confirm our findings. Second, although we appraised 53 diverse features with the ML algorithm, we did not consider additional features, such as cardiac magnetic resonance due to its retrospective nature, that may contribute to better risk prediction. Third, we did not conduct external validation to verify the robustness of our results using an independent dataset from other centers; this is our future research direction.

## Conclusions

In summary, the ML model based on preoperative and perioperative features can serve as an effective tool for the prediction of post-transplant AKI stage 3. Through the model, the risk of an individual patient with potential AKI stage 3 after HT could be identified accurately, enabling a timely intervention.

## Supplementary Information

**Additional file 1**. Feature Selection. Table S1. The Predictive Results of the model with different numbers of features.

**Additional file 2**. Machine Learning Model Comparison. Table S1. The predictive performance of different algorithms.

**Additional file 3**. Logistic Regression Model with L2 regularization.

## Data Availability

The datasets used and/or analyzed during the current study are available from the corresponding author on reasonable request.

## Abbreviations

AKI: Acute kidney injury
HT: Heart transplantation
ML: Machine learning
AUC: Area under the curve
eGFR: Estimated glomerular filtration rate
SCr: Serum creatinine
RRT: Renal replacement therapy
CKD: Chronic kidney disease
CysC: Serum cystatin C
IQR: Interquartile range
LVEF: Left ventricular ejection fraction
