# Supplementary material for: Machine learning to predict post-operative acute kidney injury stage 3 after heart transplantation
Source: BMC Cardiovasc Disord. 2022 Jun 25;22:288. doi: 10.1186/s12872-022-02721-7 (PMC9233761; doi:10.1186/s12872-022-02721-7)
Supplement: Supplementary file 1 — Additional file 1. Feature Selection. Table S1. The Predictive Results of the model with different numbers of features. [file 12872_2022_2721_MOESM1_ESM.docx]

**Additional File 1: Feature Selection.**

Generally, feature selection techniques are categorized as “filter”, “wrapper” and “embedding”. The “filter” technique describes the metric that used to evaluate the worth of feature, and the Pearson's correlation is one of the “filter” technique. It seems reasonable to select the highly correlated features, and it seems no criterion for the number of highly correlated features. Therefore, the criterion for number of highly correlated features is optional.

In this study, the criterion how we selected the features was according to the predictive performance of the model with different numbers of features in 10-fold cross-validation using Logistic regression with L2 regularization (Table S1).

Table S1. The Predictive Results of the model with different numbers of features

|  | With top 2 features | With top 3 features | With top 4 features | With top 5 features | With top 6 features | With top 8 features | With 10 features | Without feature selection |
| --- | --- | --- | --- | --- | --- | --- | --- | --- |
| AUC | 0.743 | 0.811 | 0.802 | 0.817 | **0.821** | 0.818 | 0.801 | 0.570 |

AUC, the area under the ROC curve;

After the experiments, it turned out that the logistic regression with L2 regularization with the top 6 features was the model with the highest AUC. That’s the reason why we only selected the top 6 features in the final model.
