# Supplementary material for: Machine learning to predict post-operative acute kidney injury stage 3 after heart transplantation
Source: BMC Cardiovasc Disord. 2022 Jun 25;22:288. doi: 10.1186/s12872-022-02721-7 (PMC9233761; doi:10.1186/s12872-022-02721-7)
Supplement: Supplementary file 2 — Additional file 2. Machine Learning Model Comparison. Table S1. The predictive performance of different algorithms. [file 12872_2022_2721_MOESM2_ESM.docx]

**Additional File 2:** **Machine Learning Model Comparison.**

To explore the suitable classifiers, we have selected different classifiers (e.g. naive Bayes, Random Forest, Logistic regression, Logistic regression with L2 regularization and support vector machine). We used these methods to build classifiers based on the features highly correlated with AKI stage 3. The model

Table S1. The predictive performance of different algorithms

|  | Logistic regression with L2 regularization | Logistic  regression | Random  Forest | Naïve Bayes | Support vector machine |
| --- | --- | --- | --- | --- | --- |
| AUC* | **0.821** | 0.795 | 0.712 | 0.798 | 0.816 |

The “*” was denoted that the most concerned metric in two-class problems

After the experiments, it turned out that the logistic regression with L2 regularization was the model with the highest AUC. That’s the reason why we selected this method the final model.

All machine learning experiments (e.g. feature selection, model building, and model evaluation) were implemented in WEKA 3.8.
