# Supplementary material for: Machine learning to predict post-operative acute kidney injury stage 3 after heart transplantation
Source: BMC Cardiovasc Disord. 2022 Jun 25;22:288. doi: 10.1186/s12872-022-02721-7 (PMC9233761; doi:10.1186/s12872-022-02721-7)
Supplement: Supplementary file 3 — Additional file 3. Logistic Regression Model with L2 regularization. [file 12872_2022_2721_MOESM3_ESM.docx]

**Additional File 3:** **Logistic Regression Model with L2 regularization**

The model of Logistic regression without regulization:

$log\left( \frac{p_{i}}{1-p_{i}} \right)= \beta_{0}+\beta_{1}X_{i}$ (1)

The model could be rewritten as:

$p_{i}=f(X_{i};\beta_{0};\beta_{1})= \frac{exp(\beta_{0}+\beta_{1}X_{i})}{1+exp(\beta_{0}+\beta_{1}X_{i})}$ (2)

Consider an experiment with Yi in $\{0, 1\}$ for $i=1,2, .. , n$ and with each sample $X_{i}$ available.

The likelihood of the experiment is then:

$\prod_{i=1}^{n} {[P(Y_{i}=1|X_{i})]}^{y_{i}}{[P(Y_{i}=0|X_{i})]}^{1-y_{i}}$ (3)

The log-likelihood is found to be:

$L(Y=y, X;\beta)=\sum_{i=1}^{n} y_{i}\{X_{i}\beta-log(1+exp{(X}_{i}\beta)\}$ (4)

In Logistic regression with L2 regularization, we added a ridge penalty in the log-likelihood, then we have:

$$L^{pen}\left( Y, X; \beta, \lambda\right)= L\left( Y, X; \beta, \lambda\right)- \lambda|| \beta||_{2}^{2}$$

$= \sum_{i=1}^{n} y_{i}\{X_{i}\beta-log(1+exp{(X}_{i}\beta)\} - \beta^{T}\beta$(5)
